# Policy-Relevant Attitudes Toward COVID-19 Vaccination: Associations With Demography, Health Risk, and Social and Political Factors

**DOI:** 10.3389/fpubh.2021.671896

**Published:** 2021-07-06

**Authors:** Katharina T. Paul, Jakob-Moritz Eberl, Julia Partheymüller

**Affiliations:** ^1^Department of Political Science, University of Vienna, Vienna, Austria; ^2^Department of Communication, University of Vienna, Vienna, Austria; ^3^Vienna Centre for Electoral Research, University of Vienna, Vienna, Austria

**Keywords:** vaccination, policy&politics, attitudes, COVID-19, Austria, survey, mandatory vaccination policy

## Abstract

**Background:** Vaccination is considered to be a key public health intervention to end the COVID-19 pandemic. Yet, the success of the intervention is contingent on attitudes toward vaccination and the design of vaccination policies.

**Methods:** We conduct cross-sectional analyses of policy-relevant attitudes toward COVID-19 vaccination using survey data of a representative sample of Austrian residents collected by the Austrian Corona Panel Project (ACPP). As outcomes, we examine the individual readiness to get vaccinated, the support for compulsory vaccinations, and the preference for making the vaccine available free of charge. The independent variables include demographics, objective and perceived health risks, and social and political factors.

**Results:** Although there is broad public support for making the vaccine available free of charge, vaccine hesitancy and the opposition to a vaccine mandate are widespread. The protective function of the vaccine for the individual only motivates limited support for vaccinations. Opposition to COVID-19 vaccination also stems from a lack of sense of community and an ongoing politicization of the issue through conspiracy theories and party politics.

**Conclusion:** We propose that overcoming the inherent free-rider problem of achieving sufficiently high vaccination rates poses a potential dilemma for policymakers: Given the politicized nature of the issue, they may find themselves having to choose between making vaccinations compulsory at political costs and a lingering pandemic at high costs for public health and the economy. We propose that promoting a sense of community and addressing potential practical constraints will be key in designing an effective COVID-19 vaccination policy.

## Introduction

There is widespread agreement that vaccines are one of the most effective forms of public health intervention. Yet, public support for vaccinations is a growing concern worldwide notwithstanding the ongoing COVID-19 pandemic (1). Current research suggests that the acceptance of COVID-19 vaccination is too low in many Western societies (2), leading some governments to consider mandatory vaccination, an unpopular policy instrument that typically generates political costs (3, 4) and resistance (5). As a result, policymakers find themselves in search of effective instruments to address the pandemic while keeping political costs low. In turn, this dilemma requires a new approach that moves beyond a mere focus on attitudes toward vaccination and instead also looks at attitudes toward relevant policies: In this study, we assess not only individual readiness to get vaccinated but also support for compulsory vaccinations and preferences for making the vaccine available free of charge. We do so specifically by reporting findings from the early stage of the pandemic (May 2020) based on a representative survey study, the Austrian Corona Panel Project (ACPP). The purpose of the analysis is to provide additional insights on political support for (or resistance to) emerging policies addressing COVID-19 that can inform policy design.

### Previous Research and Expectations

Existing social science and behavioral research has identified a broad range of factors that affect attitudes toward vaccinations (6). The predictors that this scholarship has identified can be grouped into three main categories: sociodemographic variables, objective and subjective health risk, as well as more general social and political factors, including collective responsibility and politicization.

First, sociodemographic variables, such as age, gender, education, and income, have received particular attention (7). More generally, surveys point to older age groups being overall more confident in vaccines and more willing to get vaccinated as hesitancy may diminish as experience with vaccines accumulates (8, 9). As for gender, studies have explained lower vaccine uptake among women with women's lower social support and a health-care-provider bias against them (8). Regarding education, the traditional assumption that vaccine rejection is determined by a lack of information is not supported unequivocally: On the one hand, vaccination campaigns in the US that included educational programs tended to obtain higher vaccine uptake compared to others (10). Conversely, research points to conscious and informed parental decisions not to vaccinate their children [see (11) for the MMR vaccine in the UK] and to increasing rates of vaccine hesitancy in highly educated communities (10, 12, 13). Finally, previous studies have also shown that financial factors such as anticipated costs and income status may present practical constraints to vaccine uptake (14, 15). Concerning other vaccine-related attitudes, such as support for compulsory vaccination, mean comparisons between individuals' type of employment showed no significant group differences (16).

Second, vaccine-related attitudes are also influenced by subjective and objective health risks. In fact, previous studies have reported respondents to provide reasoned arguments for rejecting vaccination, based mainly on the assessment of disease threat vs. potential vaccination risks, particularly among elderly respondents with chronic diseases (17, 18). In such cases, vaccines are the victims of their own success as they have reduced the prevalence, visibility, and perceived threat of infectious diseases. Psychological research captures this resultant decreasing willingness to get vaccinated as complacency (14). Conversely, research has also shown perceived severity of the disease and individuals' vulnerability to it to positively influence vaccine uptake [e.g., (19) and support for mandatory vaccination (20)].

Third, a number of social and political factors play a key role. In particular, Betsch et al. (14) argue that a sense of collective responsibility and a sense of community may increase one's willingness to protect others by means of getting vaccinated. However, empirical findings in this regard remain mixed (21). Findings are much clearer when it comes to trust in institutions (22–24), also on how it correlates with support for compulsory vaccination policies (16, 25). In fact, research suggests that the issue of vaccination is strongly politicized and distrust is actively being sown (26). Among others, medical and vaccine-related conspiracy theories (27) are undermining trust in science and health authorities (28). Populist party leaders, in particular, have been argued to try to profit from said politicization (29), making use of a vocabulary of “medical populism,” ranging from a simplification of the pandemic to being a source of misinformation (30, 31). Kennedy (32) similarly finds that those who vote for populist parties are more likely to believe that vaccines are not important, safe and/or effective.

Given these previous findings, more detailed analyses are needed as it remains unclear whether some of the findings may be specific to circumstances, particular diseases/vaccines, and measurement. Facing the current pandemic and the anticipated difficulties in reaching sufficiently high vaccine uptake, we thus examine the relationship between attitudes toward COVID-19 vaccination and related policies and the aforementioned sociodemographic, health-related, as well as social and political factors in more details below. In the context of COVID-19, we expect associations based on age, gender, and objective health risk to be all the more relevant due to high public salience of increased health risks for older age groups, men, as well as individuals with pre-existing conditions, with all other predictors (i.e., education, income situation, perceived health risk, sense of community, conspiracy belief, and party affiliation) to behave as described for the different contexts above.

## Materials and Methods

### Setting: Austria in the Early Stage of the COVID-19 Pandemic

We choose Austria as a case in which policymakers are contending with stagnating, comparatively low vaccination rates (33) despite statutory health insurance and vaccines being offered free at the point of care. Moreover, in the absence of vaccination reminder systems, direct incentives, or vaccine mandates, the Austrian immunization program remains fragmented. Beyond these systemic issues, large-scale surveys reveal that there is only limited trust in the effectiveness of vaccines, positioning Austria at the lower end of the scale, ranking national populations according to their vaccine confidence (34).

At the time of data collection, COVID-19 case numbers were low following a first wave of infections and an early countrywide lockdown. As vaccination was not available yet at the time, these conditions make for a useful baseline study of policy-relevant attitudes that we intend to study longitudinally.

### Study Design

We examine the attitudes toward COVID-19 vaccination and related policies in Austria using survey data collected by the Austrian Corona Panel Project (ACPP) that is publicly available via the Austrian Social Science Data Archive (AUSSDA). The data collection of the multi-wave online panel survey started in March 2020. A representative sample of about 1,500 Austrian residents aged older than 14 years was drawn based on quotas from a major online access panel (certified under ISO 20252). Respondents were interviewed at regular intervals, with drop-outs being compensated for by the recruitment of fresh respondents. In addition, post-stratification weights are available to match the sample distributions with population targets (for further details on the dataset, see (35). We make use of the ninth wave of the ACPP surveys (field period: 23-27/5/2020; *N* = 1,502) that included a module on policy-relevant attitudes toward a *hypothetical* COVID-19 vaccination.

### Measurement and Variables

The module on policy-relevant attitudes toward a COVID-19 vaccination covers three different aspects that we treat as outcome variables: (i) individual readiness to get vaccinated, (ii) support for compulsory vaccination, and (iii) support for free-of-charge vaccination. We asked respondents to indicate to what extent they agreed or disagreed with one of the following statements: “Once a COVID-19 vaccine becomes available,.” (a) I will get vaccinated as soon as possible, (b) there should be a compulsory vaccination for everyone, and (c) the vaccine should be provided free of charge. The responses were recorded on a five-point-scale reaching from 1 “completely agree” to 5 “completely disagree.” The responses serve as outcome variables in our subsequent analyses.

In our analyses, we focus on three groups of predictors that may inform attitudes toward a COVID-19 vaccination. First, we examine key demographics, studying how vaccination attitudes differ across age, gender, education, and income groups. Specifically, we compare the attitudes across three age groups (<30, 31–65, >65 years), men and women, and three educational levels (low, medium, high). As objective income measures often suffer from high levels of non-response, we rely on a subjective assessment of the respondent's financial situation (difficult to get by, average, doing well).

Second, we examine the role of the objective and perceived health risks to assess to what degree risk perception and perceptions of the individual protective function of the vaccine affects support for the vaccine. In the survey, respondents were asked to indicate whether they suffered from any of the following pre-existing conditions: cardiovascular diseases, diabetes, hepatitis B, chronic obstructive pulmonary disease, chronic kidney failure, and/or cancer. We compare those with pre-existing conditions to those without. In addition, we also make use of a measure of the self-assessed individual health risk created by SARS-CoV-2 grouped in three categories (1 “very/rather low,” 2 “medium” to 3 “very/rather high”).

Finally, we also take into account social and political factors. We measure what we label as sense of community in fighting the COVID-19 pandemic with an additive index created from three items (see Appendix A for question wording). For ease of representation, we group the index scores ranging from 0 to 100 in three equally spaced categories (<34 “very/rather low,” 34–66 “medium,” >66 “very/rather high”). To capture the politicization of the issue, we include measures for conspiracy belief and party affiliation. Conspiracy belief is measured using a binary indicator that was created based on two statements, namely, whether the vaccine against the coronavirus has already been developed, but (a) is being held back by large pharmaceutical companies and (b) is being held back by the government. When respondents reported being very or quite certain that one of the two statements is true, respondents were categorized as prone to conspiracy beliefs. To measure party affiliation, we include the reported vote recall from the last national election. From the ideological left to the right, we distinguish between the Green party, the center-left social democratic party (SPÖ), the liberal party Neos, the centre-right people's party (ÖVP), and the populist-right Freedom Party (FPÖ). The last three parties mentioned here can also be considered as overall critical toward state intervention. Voters of smaller parties, non-voters, and non-responders are summarized in the “Others” category.

### Statistical Analysis

The statistical analyses proceed in three steps: First, we explore the descriptive distributions of the measures capturing the policy-relevant attitudes toward a COVID-19 vaccination. Then, we evaluate the unadjusted bivariate relationships between these attitudes and the three groups of predictors as described above. Finally, we conduct a multivariate linear regression (OLS) analysis including all three groups of factors simultaneously. We do so to account for mutual confounding effects and to assess the relative importance of each group of predictors. We apply listwise deletion of missing values which leaves us with a consistent basis of 1,301 respondents with full records on all variables as a basis for the present analysis.

## Results

### Attitudes Toward COVID-19 Vaccination

**Figure 1:**
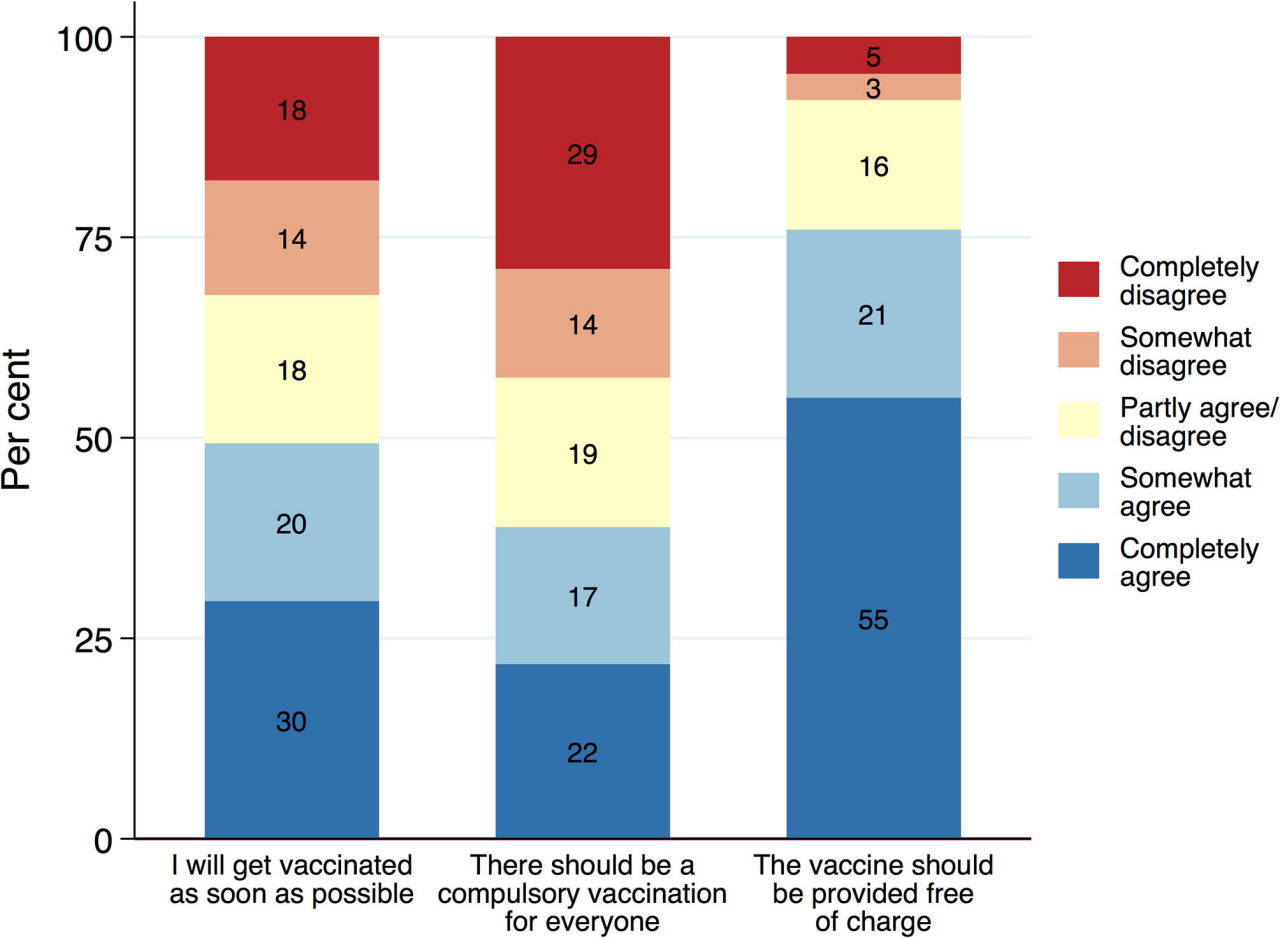
Attitudes toward a COVID-19 vaccination. ACPP data (*N* = 1,301; 23–27 May 2020; weighted).

The three different measures of attitudes toward a COVID-19 vaccination show distinct response distributions (Figure 1). The results show that, although a substantial share of the population is willing to get vaccinated as soon as possible, vaccine hesitancy is nevertheless a wide-spread phenomenon at the same time: While about 50% of the resident population agree completely or somewhat to getting vaccinated as soon as the vaccine becomes available, 32% disagree with this statement and would rather delay or not get vaccinated instead. Eighteen percentage appear to be undecided or ambivalent and choose the middle category of the scale. Estimates for herd immunity suggest a threshold of at least 67% acquiring immunity either through infection or immunization (36). Against this benchmark, the measured readiness to get vaccinated appears somewhat too low. Hence, there is a possibility that voluntary vaccinations alone might not suffice to reduce the spread of the virus drastically enough.

Facing high levels of vaccine hesitancy, policymakers across the globe are considering making COVID-19 vaccinations mandatory. The support for compulsory vaccination, however, is low and the opposition to it considerable. Only 39% of the population agree completely or somewhat with the statement that there should be a compulsory vaccination. Forty three percentage, however, disagree with the idea of making COVID-19 vaccination compulsory, of which 29% disagree strongly. Compulsory vaccinations, thus, are fairly unpopular. Interestingly, the idea of making a vaccine available free of charge, in contrast, finds broad support among the public. A total of 76% agree that the vaccine should be made available free of charge, whereas only a very small minority of 8% opposes this idea. While people seem very supportive of publicly funding vaccination - reflecting the institutions of the welfare state - this may not necessarily mean they are willing to get vaccinated themselves, perhaps preferring to benefit indirectly from vaccine-induced herd immunity without having to bear the risk of side-effects. This could point to an effect that behavioral research has labeled calculation (14) or what we would consider a classical free-rider problem.

A common solution to the free-rider problem in social policy is that the government imposes a fair burden-sharing; in the case of vaccination, this would amount to making the vaccination compulsory for everyone. Our study, however, reveals considerable opposition to such a policy measure. Policymakers are likely to face strong opposition, significant public backlash and potentially civil disobedience if they were to pursue such a strategy. Opting for such a measure without sufficient public support might even be counterproductive: it could undermine political trust and lead to reactance, and thereby reduce willingness to get vaccinated. Policymaking will thus form a careful balancing act between, on the one hand, assessing the political costs of unpopular, controversial policies such as vaccine mandates (37), and, on the other hand, addressing potentially low vaccine uptake in other, less intrusive ways (38).

### The Role of Demographics, Health Risks, and Social and Political Factors

**Figure 2:**
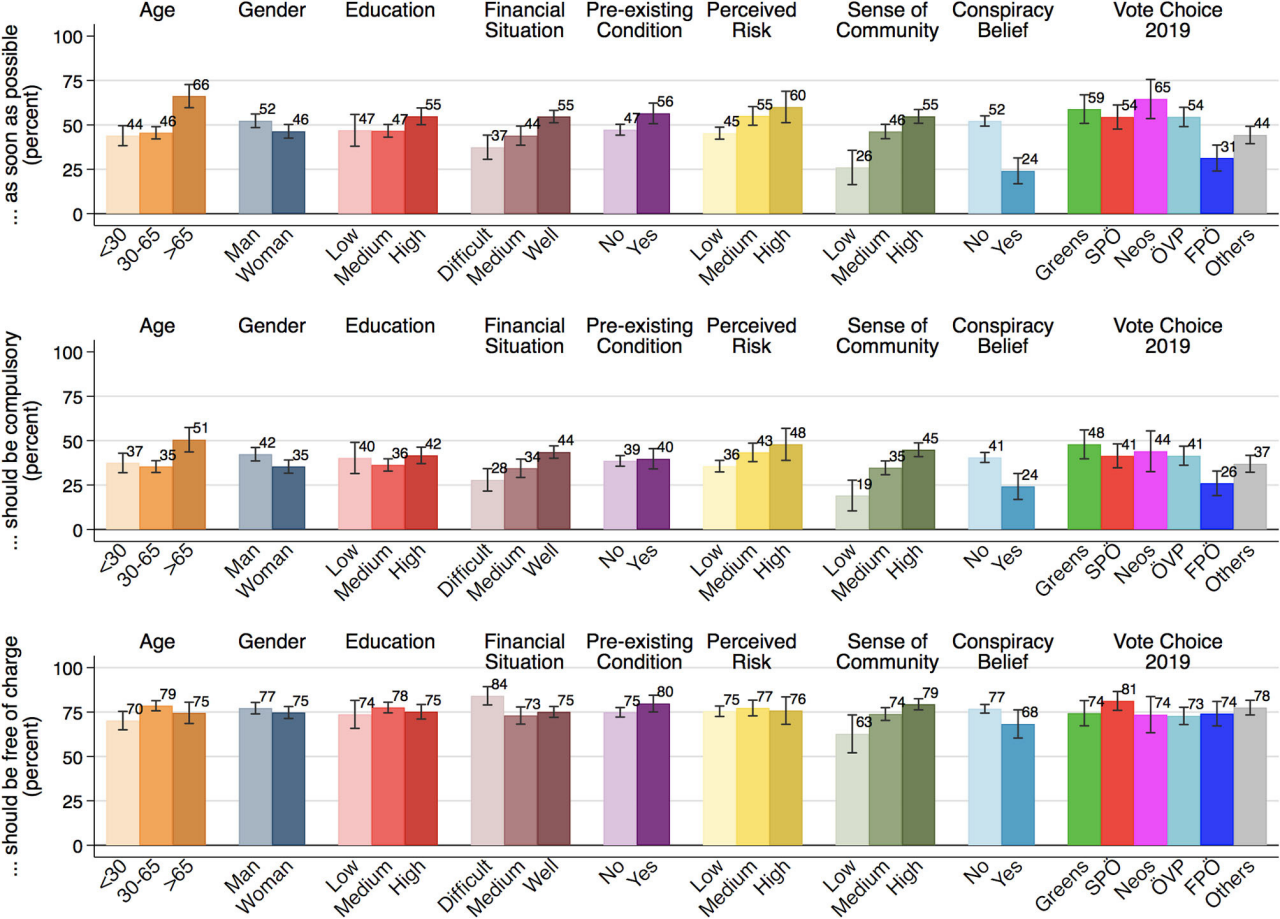
The role of socio-demographics, health risk, social cohesion, and politics. The bars show the percentages of the categories “completely agree” and “somewhat agree” combined, alongside 95% confidence intervals (ACPP data; *N* = 1,301; 23–27 May 2020; weighted).

Next, we study vaccination attitudes across social groups based on socio-demographic information, perceived and objective health risks, and social and political factors (Figure 2). We observe similar patterns for vaccine hesitancy and the opposition to compulsory vaccinations, whereas the free-of-charge vaccinations display a distinct pattern. The correlation between the willingness to get vaccinated and support for compulsory vaccination is fairly high (*r* = 0.77, *p* <0.001). This correlation is considerably higher than that with the support for making the vaccination available free of charge (*r* = 0.24, *p* < 0.001).

Concerning vaccine hesitancy and compulsory vaccination, we observe systematic differences across demographic groups: Elderly respondents aged older than 65 are more willing to get vaccinated and also are more likely to support compulsory vaccinations. Likewise, men are more supportive of getting vaccinated as well as compulsory vaccination. Interestingly, we hardly see any differences across educational groups, implying that the opposition to vaccinations is less driven by a lack of understanding or information on vaccinations. The readiness to get vaccinated is marginally higher for those with pre-existing health conditions, but we hardly see any difference in the support for compulsory vaccinations. Moreover, financially well-off people are more in favor of vaccinations than those groups experiencing difficulties in getting by. At the same time, people who are facing financial difficulties are the most in favor of making the vaccine available free of charge. Yet as we show further below, the association vanishes when controlling for other variables.

Opposition to COVID-19 vaccination springs, in particular, from a lack of sense of community and politicization. We find that the willingness to get vaccinated is lowest among those with little sense of community, believers in conspiracy theories, and the supporters of the populist-right FPÖ. These same groups also display greater opposition to compulsory vaccination. Those with a weak sense of community are also somewhat more skeptical than others of providing the vaccine free-of-charge. This is, however, not true for the supporters of the populist-right who are equally likely as the supporters of other parties to be in favor of free vaccinations.

**Figure 3:**
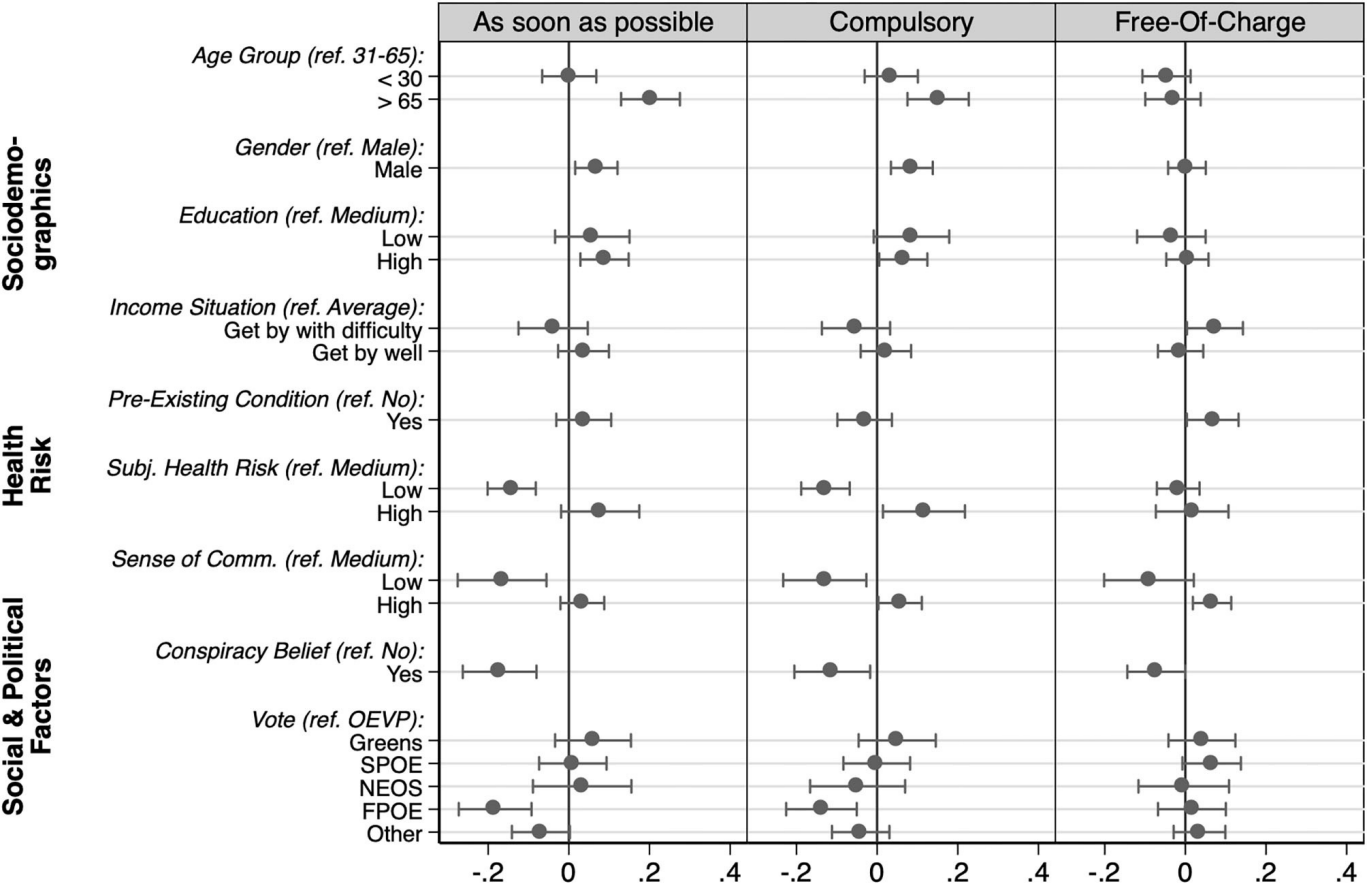
Multivariate analysis. Displayed are the unstandardized coefficients from OLS regression alongside 95% confidence intervals. Each model includes all listed predictors simultaneously (*N* = 1,301). The corresponding estimation tables are included in Appendix B.

To account for mutual confounding effects and to evaluate the relative importance of each of these factors, we conducted a multivariate regression analysis. Figure 3 visualizes the estimated coefficients. The results mostly confirm the observed bivariate associations. Even when controlling for the perceived individual health risk, sociodemographic factors such as age and gender continue to play a role, with elderly and male respondents being more likely to get vaccinated and supporting making vaccinations compulsory. Pre-existing conditions, however, no longer show a significant association with the propensity to get vaccinated, when taking the perceived individual health risk into account simultaneously. Those who perceive their personal health risk to be low, show less willingness to get vaccinated and oppose the idea of compulsory vaccinations.

Much like in our first analysis discussed above, we find hardly any systematic differences across educational groups. Likewise, the differences across income groups mostly are no longer significant when controlling for other factors. The only exception is the higher support for making the vaccine available free of charge that is slightly more strongly supported by those with financial difficulties. Overall, these results suggest that education and financial constraints play only a minor role for policy-relevant attitudes toward COVID-19 vaccination.

Finally, our analysis confirms the important role of social cohesion and politicization in informing attitudes toward vaccination and policy design. A low sense of community in fighting the COVID-19 pandemic strongly reduces the individual readiness to get vaccinated and the support for making vaccinations compulsory. In addition, conspiracy beliefs and a preference for the populist-right also are strongly associated with greater vaccine hesitancy and opposing compulsory vaccinations.

## Conclusion

These findings have important implications for the potential dilemma policymakers find themselves in. Current discourse suggests that policymakers in many advanced democracies will have to decide between making COVID-19 vaccination mandatory or a lingering pandemic due to low vaccination rates. Yet low risk perception and the politicization of the policy issue by populist parties and conspiracy theorists are hardly solvable by a vaccine mandate, not least because such policy instruments typically cause even more resistance (5) which, in turn, may have detrimental effects on vaccine uptake more generally. Although our empirical study focuses on the case of Austria, we expect similar patterns to arise in other societies where vaccine hesitancy is common. Support for vaccines may also change over time. Yet, given the underlying problem of a lack of sense of community and politicization, addressing the apparent lack of trust and inducing a stronger sense of collective responsibility should take precedence over considering compulsory vaccination.

Overall, our analysis of attitudes toward COVID-19 vaccination in Austria shows that, although there is broad public support for making the vaccine available free of charge, vaccine hesitancy and opposition to compulsory vaccination are widespread. The latter two are strongly interrelated and share common predictors, whereas making the vaccine available free of charge appears to be widely popular and is equally supported across most groups.

For vaccine hesitancy and the opposition to compulsory vaccinations, we find strong associations with all three groups of predictors – sociodemographics, health risks, and social and political factors. Most notably, older and male respondents were found to be more willing to get vaccinated and more supportive of compulsory vaccinations. In contrast, those perceiving a low health risk, feeling little sense of community in fighting the pandemic, adhering to conspiracy beliefs, and supporters of the populist-right were most strongly opposed to COVID-19 vaccination. We found little systematic patterns for education, income, and pre-existing conditions, most notably when controlling for other factors.

The contrast between widespread support for free-of-charge vaccinations and vaccine hesitancy or opposition to vaccine mandates could suggest an underlying free-rider problem where everyone is in favor of the collective benefits of the vaccine, but hesitant in the face of real or perceived individual costs. The protective function of the vaccine for the individual provides one important motive for why people support vaccination. It does not appear to be universal enough, though, to achieve sufficiently high vaccination rates to protect those who might underestimate their personal health risk or cannot get vaccinated for some reason. It is well-known that this problem can be overcome by values such as a sense of community or external enforcement, e.g., by making vaccines mandatory (39). Yet, while we find evidence that a sense of collective responsibility can motivate vaccination (14), we also see that politicization can undermine the “we-spirit” in fighting the pandemic jointly as well as the support for external enforcement via vaccine mandates.

## Data Availability Statement

The datasets presented in this study can be found in online repositories. The names of the repository/repositories and accession numbers can be found below: The data from the Austrian Corona Panel Project that support the findings of this study are openly available via AUSSDA (The Austrian Social Science Data Archive) at https://doi.org/10.11587/28KQNS.

## Ethics Statement

Ethical review and approval was not required for the study on human participants in accordance with the local legislation and institutional requirements. The patients/participants provided their written informed consent to participate in this study.

## Author Contributions

KP led the general design and setup of the paper, contributed to the analysis, and writing up and discussion of the pertinent literature. J-ME and JP were involved in survey design and data management and led the data analysis. All authors contributed to the article and approved the submitted version.

## Conflict of Interest

The authors declare that the research was conducted in the absence of any commercial or financial relationships that could be construed as a potential conflict of interest.
