# Supplementary material for: Policy-Relevant Attitudes Toward COVID-19 Vaccination: Associations With Demography, Health Risk, and Social and Political Factors
Source: Front Public Health. 2021 Jul 6;9:671896. doi: 10.3389/fpubh.2021.671896 (PMC8290156; doi:10.3389/fpubh.2021.671896)
Supplement: Supplementary file 1 [file Data_Sheet_1.docx]

**Appendix / Supplementary Material**

1. **Question wording**

**Policy-Relevant Attitudes Towards COVID-19 Vaccination**

Please indicate whether you completely, somewhat or partly agree/disagree with each statement. If there is a vaccine for the coronavirus...

- 1. ...I'll get vaccinated as soon as possible.
  2. ...there should be a compulsory vaccination for everyone.
  3. ...the vaccine should be provided free of charge

*Matrix labels:*

- *1 = completely agree*
- *2 = somewhat agree*
- *3 = partly agree/disagree*
- *4 = somewhat disagree*
- *5 = completely disagree*
- *don't know [88]*
- *no answer [99]*

**Gender**

What is your gender? (single mention)

1. Male
2. Female
3. diverse
4. no answer [99]

**Age**

What year were you born? (Single mention, number input)

1. Year of birth: <...>

**Education**

What is the highest level of education or training you have achieved? (Single mention)

1. Elementary school or less
2. Lower secondary school (Hauptschule or AHS)
3. Polytechnic, BMS (technical school, e.g. HASCH)
4. Apprenticeship, vocational school
5. Upper secondary school (AHS with Matura)
6. Higher secondary vocational school (BHS with Matura; e.g. HTL, HAK, HBLA etc.)
7. University related teaching institution or college
8. Bachelor
9. Magister / Master / Graduate Engineer / University of Applied Sciences
10. Doctor / PhD
11. no answer [99]

**Income Situation**

How do you assess the financial situation of your household in February 2020? With the household income in February 2020, were you able to... (single mention)

1. get by very well [1]
2. get by well [2]
3. neither well nor difficult [3]
4. get by with difficulty [4]
5. get by only with great difficulty [5]
6. don't know [88]
7. no answer [99]

**Pre-Existing Condition**

Are you suffering from one or more of the following diseases?

Cardiovascular disease, diabetes, hepatitis B, chronic obstructive pulmonary disease, chronic renal failure, cancer (single mention)

1. Yes
2. Yes, several
3. No
4. no answer [99]

**Perceived Health Risk**

How great do you estimate the health risk posed by the coronavirus to you personally and to the Austrian population?

1. for me personally
2. for the Austrian population

*Matrix labels:*

- *1 = very large*
- *2 = large*
- *3 = average*
- *4 = small*
- *5 = very small*
- *no answer [99]*

**Sense of Community**

In general, how much do the following statements apply to the mood in our society?

1. We are all doing our best to overcome the crisis situation.
2. Most agree that cohesion is important in the current crisis situation.
3. In the current situation, we are moving together to protect the weak.

*Matrix labels:*

- *1 = completely apply*
- *2 = somewhat apply*
- *3 = neiter/nor*
- *4 = don’t apply somewhat*
- *5 = don’t apply at all*
- *don't know [88]*
- *no answer [99]*

**Conspiracy Belief**

To what extent do you consider the following statements to be true or false when you think of the coronavirus?

1. The vaccine against the coronavirus has already been developed, but is being held back by large pharmaceutical companies.
2. The vaccine against the coronavirus has already been developed, but is being held back by the government.

*Matrix labels:*

- *1 = Very certain that this is false*
- *2 = Rather certain that this is false*
- *3 = Unsure, whether that is true or false*
- *4 = Rather certain that this is true*
- *5 = Very certain that this is true*

**Vote Choice**

Which party did you vote for in the last national election on 29 September 2019? (single mention)

1. ÖVP/ List Kurz
2. SPÖ
3. FPÖ
4. Greens
5. Neos
6. List JETZT
7. KPÖ
8. Other party
9. Invalid
10. Did not vote
11. Did not have the right to vote
12. no answer [99]
13. **Estimation tables**

**Table B1: Multivariate analysis**

|  | (1)  As soon as possible | | (2)  Compulsory | | (3)  Free-Of-Charge | |
| --- | --- | --- | --- | --- | --- | --- |
| **Sociodemographics** |  |  |  |  |  |  |
| *Age Group (ref. 31-65)* |  |  |  |  |  |  |
| < 30 | -0.046 | (0.098) | 0.157 | (0.104) | -0.133 | (0.077) |
| > 65 | 0.554*** | (0.110) | 0.502*** | (0.117) | -0.074 | (0.087) |
| *Gender (ref. Female)* |  |  |  |  |  |  |
| Male | 0.254** | (0.078) | 0.306*** | (0.083) | 0.014 | (0.061) |
| *Education (ref. Medium)* |  |  |  |  |  |  |
| Low | 0.263 | (0.136) | 0.272 | (0.144) | -0.114 | (0.107) |
| High | 0.289** | (0.089) | 0.162 | (0.095) | 0.013 | (0.070) |
| *Income Situation (ref. Average)* |  |  |  |  |  |  |
| Get by with difficulty | -0.183 | (0.125) | -0.155 | (0.133) | 0.250* | (0.099) |
| Get by well | 0.130 | (0.092) | 0.085 | (0.098) | -0.042 | (0.073) |
| **Health Risk** |  |  |  |  |  |  |
| *Pre-Existing Condition (ref. No)* |  |  |  |  |  |  |
| Yes | 0.107 | (0.099) | -0.056 | (0.106) | 0.216** | (0.079) |
| *Subj. Health Risk (ref. Medium)* |  |  |  |  |  |  |
| Low | -0.578*** | (0.088) | -0.560*** | (0.094) | -0.095 | (0.070) |
| High | 0.222 | (0.148) | 0.296 | (0.158) | 0.062 | (0.117) |
| **Social & Political Factors** |  |  |  |  |  |  |
| *Sense of Comm. (ref. Medium)* |  |  |  |  |  |  |
| Low | -0.553*** | (0.164) | -0.607*** | (0.175) | -0.406** | (0.129) |
| High | 0.133 | (0.079) | 0.165 | (0.085) | 0.140* | (0.063) |
| *Conspiracy Belief (ref. No)* |  |  |  |  |  |  |
| Yes | -0.570*** | (0.127) | -0.451*** | (0.136) | -0.225* | (0.101) |
| *Vote (ref. OEVP)* |  |  |  |  |  |  |
| Greens | 0.116 | (0.139) | 0.220 | (0.149) | 0.189 | (0.110) |
| SPOE | 0.007 | (0.122) | 0.062 | (0.129) | 0.200* | (0.096) |
| Neos | -0.033 | (0.178) | -0.115 | (0.190) | 0.005 | (0.141) |
| FPOE | -0.572*** | (0.135) | -0.470** | (0.144) | 0.007 | (0.107) |
| Other | -0.275** | (0.105) | -0.144 | (0.112) | 0.109 | (0.083) |
| _cons | 2.388*** | (0.137) | 1.920*** | (0.146) | 3.129*** | (0.108) |
| *N* | 1,301 | | 1,301 | | 1,301 | |
| R^2^ | 0.155 | | 0.116 | | 0.049 | |

*Note*: Entries are unstandardised coefficients from OLS regression. Standard errors in parentheses. * p<.05, **p<.01, *** p<.001.
